# Insanity Cured by the Removal of Carious Teeth
*For this interesting case we are indebted to Dr. J. R. Ralston of Hopeville P.O. Tenn., for whom it was described by Dr. Perry. Dr. Ralston writes as follows:"Dr. Gorgas:—All the parties mentioned in this article are well known to myself. I have known them all my life, with the exception of Dr. Perry, whom I have known for several years. He is a pious and truthful man. If you think best you can publish Dr. Perry's letter in the *American Journal of Dental Science*. The circumstances as stated are well known to every person in this county."J. R. Ralston.


**Published:** 1869-06

**Authors:** W. T. Perry

**Affiliations:** of Maury Co., Tenn.


### ARTICLE. II.

### *Insanity Cured by the Removal of Carious Teeth.**

By W. T. Perry, M. D., of Maury Co., Tenn.

In compliance with promise and inclination, I submit for your disposal such facts as I have in reference to the following case, which came under my professional care and observation a few months previous to the recovery of the patient.

Mrs. B., aged 35, of nervous sanguine temperament and good physical strength and form, has been married twice but has never borne children, and is now living with her surviving husband who resided in an adjoining state at the time of the first occurrence of insanity with his wife. From him I learn that her general health previous to attack had ordinarily been good, with the exception of occasional slight attacks of menorrhagia. She has also suffered more or less with indigestion, which at times became troublesome in its effects, being followed by colic, neuralgic affections and occasional afflictive spells of odontalgia, the result probably of decaying teeth.

In her general mental and moral characteristics, she was a gentle, kind, religious lady of good social qualities, pleasant and affable, and much loved and esteemed by her acquaintances, but! how changed the scene! how altered the mental and moral characteristics of this amiable lady when "reason was dethroned," and the beauty and symmetry of a well regulated mental and moral constitution was spoiled by this grave mysterious affection. This disease first made its appearance in the form of slight mental aberations, evinced by her lavish kindness in giving away her clothing and other property to the amount of nearly all she had. This was soon succeeded by an exaltation of the cerebral functions with increased perversion amounting to *Raving*

---

* For this interesting case we are indebted to Dr. J. R. Ralston of Hopeville P.O. Tenn., for whom it was described by Dr. Perry. Dr. Ralston writes as follows:

"Dr. Gorgas:—All the parties mentioned in this article are well known to myself. I have known them all my life, with the exception of Dr. Perry, whom I have known for several years. He is a pious and truthful man. If you think best you can publish Dr. Perry's letter in the *American Journal of Dental Science.* The circumstances as stated are well known to every person in this county."

J. R. Ralston.

*Mania*, which became so persistent and violent as to render seclusion and confinement necessary. She was accordingly taken to an *asylum for the insane* for treatment, at which place she remained for nearly two years, realizing no decided improvement or change in her mental lesion.

Her case being considered by her husband and friends as confirmed and unpromising of favorable results, she was removed to this community, and kept in close confinement on account of paroxysmal boisterous, destructive disposition. It was under these latter circumstances that I learned she was afflicted with sore and swelled gums, and at times suffered much with neuralgia involving the the teeth, face and contiguous parts. Observing that she had quite a number of decayed teeth and unremoved fangs of teeth, I suggested their removal by extraction as a means of alleviating her sufferings and possibly mitigating her mental disease. This was accordingly done by removing a portion at a time at intervals of a week or more (just as we could prevail upon our patient to submit), which was soon followed by decided and marked improvement, both with reference to sufferings and mental disease, and finally with perfect and complete recovery.

She now attends in person to her household and domestic affairs, and for the past six months, up to this writing, has remained free from insanity.

I consider the case one of recovery after having been continuously insane for three years, and that the result of recovery was effected by the removal of carious and defective teeth, as no other remedy of a special character was used at the time.

---

